# Fabrication and In Vitro Biological Assay of Thermo-Mechanically Tuned Chitosan Reinforced Polyurethane Composites

**DOI:** 10.3390/molecules28207218

**Published:** 2023-10-22

**Authors:** Nadia Akram, Iram Shahzadi, Khalid Mahmood Zia, Muhammad Saeed, Akbar Ali, Rashad Al-Salahi, Hatem A. Abuelizz, Francis Verpoort

**Affiliations:** 1 Department of Chemistry, Government College University Faisalabad, Faisalabad 38000, Pakistan; iram.shahzadi730@gmail.com (I.S.); drkmzia@gcuf.edu.pk (K.M.Z.); msaeed@gcuf.edu.pk (M.S.); akbarali@gcuf.edu.pk (A.A.); 2 Department of Pharmaceutical Chemistry, College of Pharmacy, King Saud University, Riyadh 11451, Saudi Arabia; ralsalahi@ksu.edu.sa (R.A.-S.); habuelizz@ksu.edu.sa (H.A.A.); 3 State Key Laboratory of Advanced Technology for Materials Synthesis and Processing, Wuhan University of Technology, Wuhan 430070, China; francis@whut.edu.cn

**Keywords:** chitosan, polyurethane composites, TGA, DMA, biocompatibility

## Abstract

The progressive trend of utilizing bioactive materials constitutes diverse materials exhibiting biocompatibility. The innovative aspect of this research is the tuning of the thermo-mechanical behavior of polyurethane (PU) composites with improved biocompatibility for vibrant applications. Polycaprolactone (CAPA) Mn = 2000 g-mol^−1^ was used as a macrodiol, along with toluene diisocyanate (TDI) and hexamethylene diisocyanate (HMDI), to develop prepolymer chains, which were terminated with 1,4 butane diol (BD). The matrix was reinforced with various concentrations of chitosan (1–5 wt %). Two series of PU composites (PUT/PUH) based on aromatic and aliphatic diisocyanate were prepared by varying the hard segment (HS) ratio from 5 to 30 (wt %). The Fourier-transformed infrared (FTIR) spectroscopy showed the absence of an NCO peak at 1730 cm^−1^ in order to confirm polymer chain termination. Thermal gravimetric analysis (TGA) showed optimum weight loss up to 500 °C. Dynamic mechanical analysis (DMA) showed the complex modulus (E*) ≥ 200 MPa. The scanning electron microscope (SEM) proved the ordered structure and uniform distribution of chain extender in PU. The hemolytic activities were recorded up to 15.8 ± 1.5% for the PUH series. The optimum values for the inhibition of biofilm formation were recorded as 46.3 ± 1.8% against *E. coli* and *S. aureus* (%), which was supported by phase contrast microscopy.

## 1. Introduction

A biomaterial is a constituent of living beings, which can originate from both plants and animals and can be used directly or after processing [1]. These biomaterials are ideal candidates for numerous materials with various biomedical applications, together with utilities in other fields of interest. The biomaterials derived from polysaccharides can be categorized into two dissimilar kinds [2,3,4]. One form is derived from starch that can be obtained from wheat, corn, potato, and glycogen. The other type is obtained from chitosan, which is extracted from the external skin or shell of crustaceans [5,6,7].

There is a continuous demand for eco-friendly and cost-effective materials, which makes biomaterials a favorite candidate to fulfil this demand [8,9,10]. Among various available biopolymers, chitosan is an appropriate material to meet these challenges. The exoskeletons of crustaceans, molluscs, and insects provide amino polysaccharide chitin. It is converted into chitosan and its derivatives after deacetylation [11,12,13]. The versatility of chitosan has enhanced its opportunity to be used in various sectors, including materials, medicines, energy, etc. Chitosan (1-4)-2-amino-2-deoxy-b-D-glucan) is developed from *N*-acetyl-D-glucosamine monomers with b-1,4- glycosidic bonds. It can be attained by the deacetylation of chitin extracted from crustacean shells [14,15,16]. The biocompatibility of chitosan has been reported in terms of antibacterial, anti-inflammatory, hemostatic, and skin regenerative behavior. The amino (-NH_2_) and hydroxyl (-OH) functional groups on the molecular chains may also assist in enhancing various biological functions [17,18]. It is very important to emphasize the mode and conditions of the physico-chemical and mechanical properties of the final product. It can play a significant role in deciding the applications of these materials. Hence, the chemical alteration in the structure is considered a direct source to induce new functional properties. Its antibacterial, anti-fungal, and anti-inflammatory properties have magnified its applications on a broader scale. It has the tendency to be adjusted easily with a variety of combinations of composites [19,20].

Technological advancements have innovated multiple materials, and it is worth mentioning that these products are mostly formed with high-quality raw materials. One such promising material is polyurethane. It has a broad window of the raw materials [21,22]. This is a unique polymer that can be developed by any petroleum-based monomers, biomaterials, or a blend of both. The essential component of PU is high molecular weight structures, commonly known as macrodiols or polyols. The other components are short-chain diols or diamines, which act as chain extenders or chain terminators, along with diisocyanates. The chemical reaction between these components produces urethane linkage, which is the repeating unit of PU. The most important feature of this polymer is the development of a hard segment (HS). It is produced by the combination of diisocyanate and chain extender. The second important part is the soft segment (SS). It is developed from long-chain polyol. In the polymeric chains of PU, both these segments are arranged alternatively and are most commonly used to adjust the desired properties. Another important aspect of this vibrant polymer is the development of composites by the addition of reinforcing agents/fillers, which has added another interesting and beneficial feature in PUs [23].

Composites are absolute engineering materials meant for swift innovations in various fields, particularly in biomedical applications. The gigantic increase in the population has raised the eyebrow to keep them safe and healthy. There is a dire need for biocompatible materials for all types of usage. During situations such as pandemics, life assurance is the prime task. Hence, the probe of new biocompatible material is inevitable under such circumstances. Numerous researchers are on a quest to develop innovative biocompatible polyurethane composites. Gawlikowski et al. [24] developed in vitro biocompatible and hemocompatible PET Copolyesters. It was designed for heart-assisting devices. Cervera et al. [25] investigated the biocompatibility of polyurethane electrospun membranes. The product was based on arginine as a chain extender. Xie et al. [26] worked on the construction and biocompatibility of three-dimensional composite polyurethane scaffolds in a liquid crystal state. Alyamaç et al. [27] developed novel polyurethane foams with titanium powder and collagen for medical uses. This research has proven that scientists are striving to produce more biocompatible materials that can be used to solve various biomedical issues. The urge to develop new biocompatible materials is getting recognition in the field of polymers. Polyurethane-based nanocomposites were also developed by Baysal et al. [28] using various concentrations of organoclay (1, 3, 5, and 10 wt % in each case). Zhao and colleagues [29] synthesized a composite material with a laminated structure. It was made by layering ceramic and metal in a certain order. Li et al. [30] used semi-solid electromagnetic stirring casting to synthesize graphene nanoplatelet (GNP)-reinforced AZ91D (GNPs/AZ91D) composites. Along with their mechanical characteristics, the composite’s microstructural behavior was studied. A new nanocomposite film (Cs/FeS film) was synthesized by Shen et al. [31]. They utilized chitosan (Cs) and Bio-FeS, which were produced by Deinococcus wulumuqiensis R12 for specific food packaging. A number of chain-end functional polymers made up of poly(N-isopropylacrylamide) (PNIPAM) and 2-amino-2-deoxy-D-glucopyranose(D-glucosamine) have been synthesized by Cui et al. [32]. They have demonstrated outstanding in vitro biocompatibility. According to Yang and coworkers [33], investigations of Caprinae horn sheaths’ biological composites have revealed fascinating mechanisms of toughening and strength. In order to strengthen epoxy-matrix composites, Yang et al. [34] developed interplay hybridization of silk and carbon fibers. According to Piotrowska-Kirschling and Brzeska [35], the impact of chitosan on the chemical, structural, and morphological polyurethane/chitosan composites has been examined. Ikram et al. [36] conducted research on recent advances in chitosan/graphene-based bio-nanocomposites for energetic applications. Ji et al. [37] discovered composite sepiolite/chitosan layer-by-layer coated flexible polyurethane foams. The material showed outstanding mechanical and energy absorption characteristics for green packaging applications. Moustafa et al. [38] created rational formulations of sustainable polyurethane/chitin/rosin composites supplemented with ZnO-doped-SiO2 nanoparticles. Chitosan with polyurethane grafting as new biomaterials for controlled drug delivery was the focus of research by Mahanta et al. [39]. Maamoun and Mahmoud [40] observed the mechanical and microbial potentials of flexible polyurethane foam with chitosan.

The current research is an attempt to add a few biocompatible materials to the list. Although PU is a multidimensional polymer with numerous vibrant properties, the prime drawback associated with this polymer is the feeble condition of biostability. In the case of long-term usage, poor susceptibility to cope with biodegradation is a major concern. The conventional modes of degradation, such as hydrolysis, oxidation, metal-induced oxidation, environmental stress cracking, and enzyme-assisted degradation, are complex and expensive measures. These techniques are also responsible for the loss of mechanical behavior, leading to the material’s fracture [41,42].

Hence, there is a dire need to produce innovative biocompatible polymers with the aid of foreign reinforcing agents with controlled and reproducible physico-mechanical and biological properties in order to overcome these drawbacks. The currently available vegetation has protracted the variety of raw materials for the amplification of novel biobased macromolecular architectures. This has allowed the expansion of biobased PUs with progressive biocompatible properties. This can lead to new tunable biomedical applications. PU structures with precise tissue biomimicking, new smart shape-memory Pus, and others with adaptable properties have displayed promising biomedical applications, including wound healing. Petroleum-based raw materials and biomaterials as substitutions for biomedical implants are gradually improving. The novelty of this work is the certification of bio-derived PU composites, which can be ascertained by the incorporation of a renewable fragment of chitosan in order to boost biocompatibility. The biocompatibility is directly linked to the structural architecture and the precise ratio of thermo-mechanical properties for many biomedical applications. The right exhibition of composition, stoichiometry, thermo-mechanical, and morphological properties guarantees a perfect composite.

In order to meet these challenges in the current research, two series of PU biocomposites were prepared. For this purpose, polycaprolactone (CAPA: Mn = 2000 g-mol^−1^) was used as a macrodiol. Two diisocyanates, toluene diisocyanate (TDI) and hexamethylene diisocyanate (HMDI), were used. In the first step, polyol and diisocyanate were reacted in a three-necked reaction flask. This generated NCO-terminated pre-polymer chains, followed by the addition of i-4 butane diol as a chain extender. The individual samples were reinforced with chitosan using different (1–5 wt %) concentrations. The consistency was maintained by the addition of acetone as a solvent. By following the same procedure, twelve samples were synthesized, constituting two series of five samples of composites, in addition to a pristine sample without incorporating the chitosan. All the synthesized samples were dried and stored in a desiccator for the structural, thermo-mechanical, morphological, and in vitro biological activities.

## 2. Results and Discussion

### 2.1. Confirmation of Linkages of PU Composites by FTIR Analysis

FTIR is a conventional technique to describe the development of important polymeric linkage. Particularly, it is used to observe the development of urethane linkage in the case of PU [43,44,45]. Some of the important linkages are provided in Table 1. N-H stretching peak was observed at 1650–1550 cm^−1^, which is a characteristic of amide linkage developed in the urethane bond and is also considered the confirmatory peak for the PU. The symmetric and asymmetric stretching vibrations of the C-H group were observed in the range of 2960–2857 cm^−1^. A band for urethane C=O was observed in the range of 1740–1709 cm^−1^. CH_2_ symmetric and asymmetric peaks were observed in the range of 2882–2867 cm^−1^ and 2960–2857 cm^−1^, respectively. Similarly, bending vibrations of the N-H group were observed at 1631–1549 cm^−1^. Stretching vibration band for C-O-C was observed at 1060–1049 cm^−1^ [28].

### 2.2. Estimation of Thermal Stability of PU Composites by TGA

**Figure 1:**
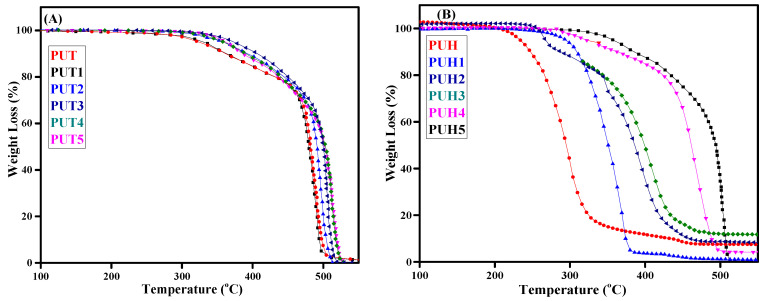
TGA curves of all the synthesized samples of PU composites (**A**) TGA curves of the PUT series (**B**) TGA curves of the PUH series.

The monomers and the final product of the polymers play a key factor in deciding the thermal stability, which is often complex as compared to a single molecule. The nature and complexity of recording the thermal behavior extend according to the components involved. This behavior is also directly related to the degradation profile of the polymers, where it is assumed to consume more fuel to degrade it or the release of toxic gases due to the breakdown of the multicomponent. The TGA thermograms of composites of the PUT and PUH series are shown in Figure 1A,B. The curves of all the composites, along with pristine samples, were arranged precisely. The multistep degradation of composites was observed in the case of both series. The data for the thermal stability at the rate of 10% weight loss and 90% weight loss are recorded in Table 1. In the case of the PUT series, the pristine sample showed thermal stability of 251 °C while the optimum thermal stability was observed ≥400 °C. Therefore, this indicates the increase of 50 °C in the thermal stability, whereas the 20 °C increase in thermal stability was observed with 90% mass loss. However, the opposite trend was observed with the PUH series, where a decrease in the thermal stability was observed in the case of aliphatic diisocyanate [44,45]. The addition of chitosan as reinforcement in the main chain resulted in an increase in the hydrogen bond length. The extended bond length in between urethane linkages was donated by polyurethane chains. The change in hydrogen bonding results in the sudden transition from a glassy state towards a rubbery state. Researchers have reported that increasing the weight of chitosan up to 20% can increase the melting temperature of polycaprolactone diols. This was attributed towards the limiting mobility of polycaprolactone diol (PCL) chains by the addition of chitosan, ultimately reducing their ordering and, finally, crystallinity. This may also result in the hindrance of the easy mobility of matrix chains. There is a probability of cavitation or discontinuity in the chain networking, leading to the obstruction of the stress transmission and the development of particle agglomeration [35].

### 2.3. Analysis of Mechanical/Viscoelastic Behavior of PU Composites by DMA

**Figure 2:**
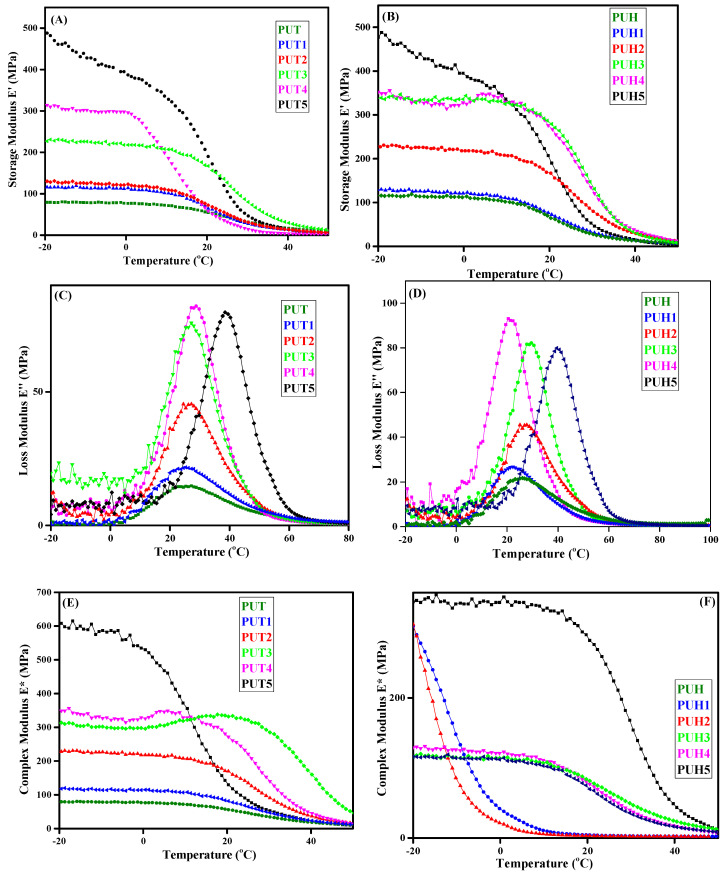
DMA curves of all the synthesized samples of PU composites (**A**) Storage modulus (E′) trend of PU composites w.r.t. temperature for the PUT series (**B**) Storage modulus (E′) trend of PU composites w.r.t. temperature for the PUH series (**C**) Loss modulus (E″) trend of PU composites w.r.t. temperature for the PUT series (**D**) Loss modulus (E″) trend of PU composites w.r.t. temperature for the PUH series (**E**) Complex modulus (E*) trend of PU composites w.r.t. temperature for the PUT series (**F**) Complex modulus (E*) trend of PU composites w.r.t. temperature for the PUH series.

The role in determining the composite’s compositional and architectural impact on the viscoelastic behavior is very crucial [2,4]. There are various parameters that have been drawn from DMA and are used to explain the mechanical behavior of the composites. Similar behavior has been evaluated for the PUT and PUH series. The relationship between storage modulus, loss modulus, and complex modulus w.r.t temperature is shown in Figure 2A–F for both the PUT and PUH series. The data of the complex modulus are reported in Table 1. The E′ evaluates the tendency of the polymeric materials to hold and store the energy of the material elastically. It is also a parameter to determine the elasticity of the material. The values of E′ decide the mechanical fate of the composites; the higher values indicate the stiffness in the chains. Figure 2A shows the E′ values of the PUT series, with the pristine sample showing lower values of storage modulus as compared to the composites with varying concentrations of chitosan. Similar behavior has also been observed in the case of the PUH series. No significant difference in pristine samples of both the PUT and PUH series was observed; however, E′ of the PUH series is slightly higher than that of the PUT series. The increases in the values of E′ were observed with the increase in HS contents. Figure 2C,D represent a relationship between temperature and loss modulus. The hump-shaped curves were observed in which the higher peak values of the hump corresponded with increased concentration of chitosan for both series. The E″ represents the viscous behavior of the material. The importance of temperature in depicting the behavior of any material cannot be denied. The ability of a material to show any particular property is highly dependent on the absorption of energy. According to the law of conservation of energy, the material cannot hold the absorbed energy forever; it certainly has to get rid of that energy. Hence, the absorption and dissipation of energy are equally significant. The absorption and dissipation of energy is dependent on several factors. One such factor is the segmental mobility of the polymers. One of the important factors in evaluating the viscoelastic behavior is the tan δ, which is obtained as a ratio of E″/E′. Figure 2E,F represent the trend of E* w.r.t temperature for both the PUT and PUH series. Similar to the behavior of E′, the increase in the values of E* was observed with the increase in the concentration of chitosan for the PUT series. However, a decrease in the values of E* for the PUH series was observed. The crosslinking, which evolved in the polymers, is the main factor in the improved mechanical behaviour of PU composites reinforced with chitosan. The chance of stable arrangement increased as a result of the networking, directly influencing the features. The formation of new arrangements may involve temperature transitions, which directly affect storage as well. The role of chitosan and its direct proportion as a reinforcing agent is crucial to all of these significant and small transformations. Polyurethane composites have enhanced mechanical behavior, according to other researchers, making them a good choice for biological applications. It is crucial to keep in mind that the chemical structures of the contributing monomers may cause networking, which might make samples brittle and significantly reduce their mechanical capabilities. The optimization in polyurethane is essential since chitosan is semicrystalline by nature. Scientists have often noticed a decline in the mechanical characteristics at higher concentrations [46].

### 2.4. Solvent Absorption Tendency of PU Composites by the Swelling Test

**Figure 3:**
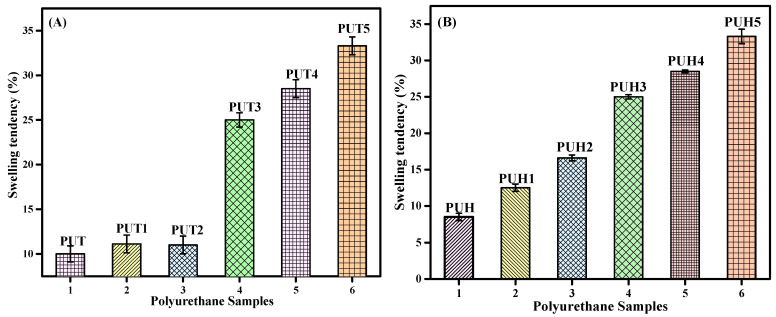
Swelling behavior of PU composites in water (**A**) Swelling behavior of the PUT series (**B**) Swelling behavior of the PUH series.

The polarity of the polymers plays a significant role in deciding the application of the polymers and their related composites, particularly those that involve the interaction with water or any other solvents [41,42,47,48,49,50]. Water, being the universal solvent, is one of the prime solvents that can show a significant impact on any kind of composite. The water absorption tendency of all the samples of the PUT and PUH series is presented in Figure 3A,B, respectively. The unique architecture of PU contains both polar and nonpolar groups in the backbone. Hence, the contribution of both HS and SS can absorb or repel water accordingly. The water absorption tendency in the case of the PUT and PUH series increased, indicating that chitosan is the main factor responsible for the absorption of water. In order to understand this effect, it is necessary to take a closer look at the polarity of each monomer involved in the formation of the composites.

### 2.5. Investigation of In Vitro Biological Activities Representing the Biocompatibility of Polyurethanes

#### 2.5.1. Hemolytic Assay of PU Composites as a Biocompatibility Test

**Figure 4:**
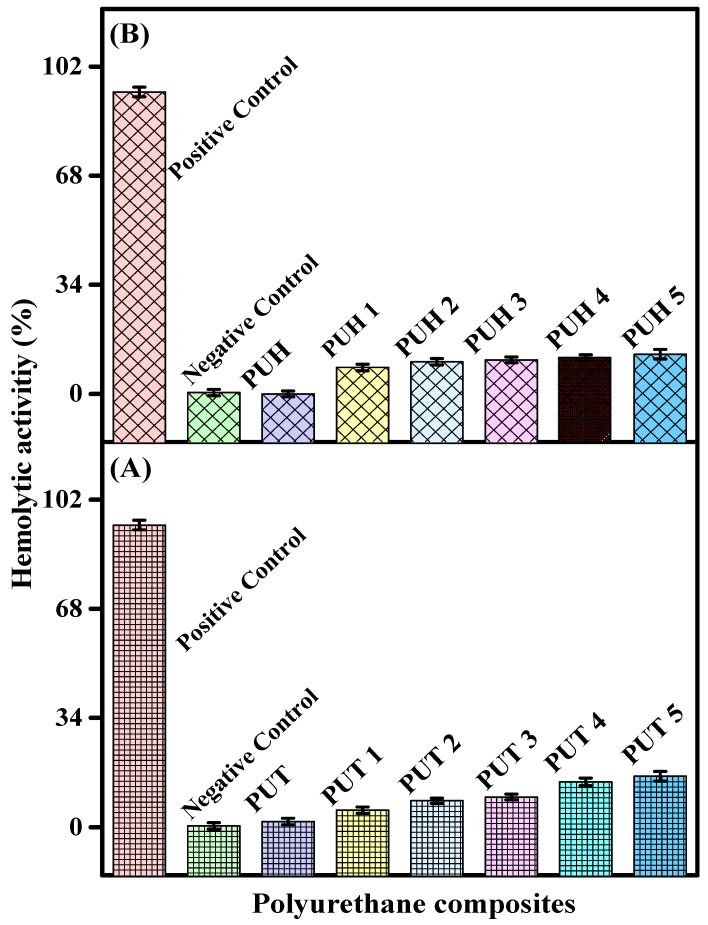
Hemolytic activity of PU composites (**A**) Hemolytic activity of the PUT series (**B**) Hemolytic activity of the PUH series.

The ever-increasing interest of researchers to utilize composites in biomedical applications has opened new windows of exploration. However, the first step towards biomedical application is the evaluation of the biocompatibility of the composites. Hemolysis is one of the authentic tests for evaluating biocompatibility, where the composites come in direct contact with red blood cells (RBCs), rupturing the cells. The data on hemolytic activity of the PUT and PUH samples are documented in Table 2. The graphical representation is shown in Figure 4. The PUT series showed hemolytic activity of 1.0 ± 0.5% for the pristine sample. The optimum hemolytic activity was observed as 12.3 ± 1.5%, whereas a similar trend was also observed for the PUH series with optimum hemolytic activity of 46.3 ± 1.8%. The data show the release of hemoglobin or the damaging of the membrane, which were compared with the positive control using triton-X 100 (94.1 ± 3.93%) as an indication of the extreme mutilation to erythrocytes (RBC). The phosphate buffer saline (PBS) was used as a negative control, which showed the values (0.41%) of lysis to erythrocytes as RBC, evaluating the least mutilation. The composites of the PUT series showed lower values of hemolytic activity as compared to the PUH series. Hence, it can be assumed that though the PU as the pristine sample is biocompatible in nature, the addition of chitosan has not compromised its hemolytic activity. All the samples of both series have shown values closer to negative control, which has endorsed that chitosan-reinforced composites can be used as biocompatible materials. Despite the fact that biocompatibility is a major problem not just for general use but also for medicinal purposes, researchers have developed a compelling case for the frequent misunderstanding of the word “biocompatibility” as it is related to material performance. Although biocompatibility is typically thought of and treated as being non-toxic, there are other factors that apply in these situations. The word “biocompatibility” is used to describe a material’s resistance to toxicity and applies to polymeric biomedical materials as well. From this important point, biocompatibility also includes the impact of the physiological environment on the functionality of the material, whether it is favorable or unfavorable [51].

#### 2.5.2. Biofilm Inhibition of PU Composites as the Biocompatibility Test

**Figure 5:**
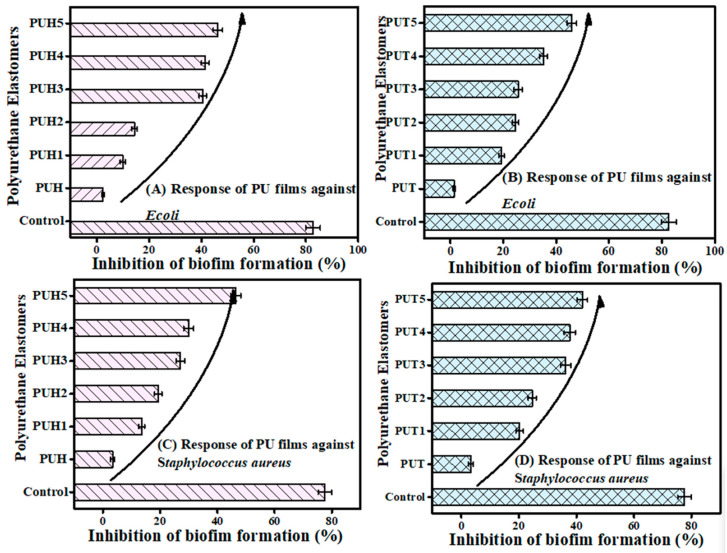
Inhibition of film formation data of PU composites; (**A**) Inhibition of film formation data of PUT against *E. coli* (**B**) Inhibition of film formation data of PUH against *E. coli* (**C**) Inhibition of film formation data of the PUT series against *S. aureus* (**D**) Inhibition of film formation data of the PUH series against *S. aureus*.

Bacterial infection is one of the most infectious factors to deteriorate the composites. Due to several varieties of the bacterial strains, the control becomes very important. The biocompatibility of the composites was evaluated using two bacterial strains. The results of biofilm inhibition as the biocompatibility factor are documented in Table 2. The biofilm inhibition activity of 77.57 ± 2.37% against *S. aureus* and 82.65 ± 2.74% against *E. coli* was observed. For both bacterial strains, the biofilm inhibition activity increased by increasing the concentration of chitosan. Hence, the strength of the biocompatibility can be improved by the addition of chitosan under optimized conditions. For the PUT series, the pristine sample showed activity of 1.4 ± 0.4 against *E. coli*, whereas for the optimum concentration of chitosan, the maximum activity was recorded as 45.8 ± 1.8%. In the case of *S. aureus*, the optimum activity of 42.0 ± 1.9 was obtained with a maximum concentration of chitosan as compared to the pristine sample of 3.3 ± 0.8. The difference in the biocompatibility of both strains was observed due to the fact that *E. coli* attacks severely. However, its infections are cured earlier. On the other hand, the strain of *S. aureus* attacks in a mild way; however, the recovery time is prolonged. Hence, a similar trend was recorded against both strains for the PUH series as well, which has shown slightly higher biocompatibility as compared to the PUT series. The graphical representation is provided in Figure 5. Due to its mucoadhesive and absorption-increasing qualities, chitosan and its derivatives were demonstrated to be good and safe options for improving mucosal and trans-mucosal administration of medications during the past 20 years. Chitosan has a positive charge and possesses the unique ability to stick to mucosal surfaces. It facilitates the interaction of the medicine with the mucus layer, covering various epithelial surfaces. Numerous studies have shown that chitosan has the ability to temporarily widen tight junctions between epithelial cells in both in vitro and in vivo analyses. This ability allows the passage of poorly absorbable macromolecules through epithelial barriers that are well-organized [51]. In addition to all these positive features, chitosan has been reported to exhibit other relevant properties, including biodegradability and biocompatibility.

#### 2.5.3. Phase Contrast Microscopic Analysis of PU Composites as the Biocompatibility Test

**Figure 6:**
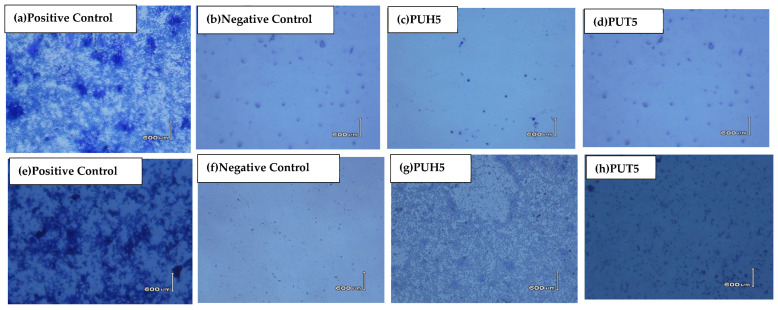
Phase contrast microscopy of PU composites (**a**) Phase contrast microscopy of positive control for *E. coli* (**b**) Phase contrast microscopy of negative control for *E. coli* (**c**) Phase contrast microscopy of PUH 5 against *E. coli* (**d**) Phase contrast microscopy of PUT 5 against *E. coli* (**e**) Phase contrast microscopy of positive control against *S. aureus* (**f**) Phase contrast microscopy of negative control against *S. aureus* (**g**) Phase contrast microscopy of PUH 5 against *S. aureus* (**h**) Phase contrast microscopy of PUT 5 against *S. aureus*.

The data of biofilm inhibition were supported microscopically using phase contrast microscopy. Some of the selected micrographs against both bacterial strains are shown in Figure 6. The negative control of the sample was provided using crystal violet, which showed a relatively less dense medium as compared to the positive control bacteria. Figure 6 of composites shows the data closely related to the positive control. The figures clearly show microbial inhibition of the bacterial strains, providing evidence that the biocomposites have shown significant antibacterial activity and have endorsed the data of biofilm inhibition activity.

### 2.6. Scanning Electron Microscopy (SEM)

**Figure 7:**
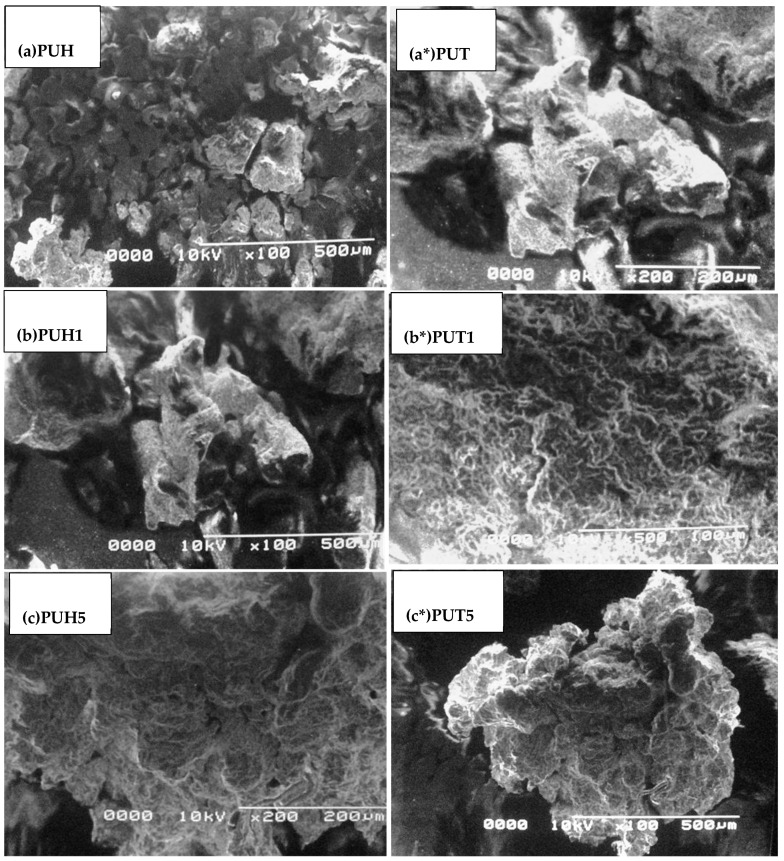
SEM morphology of PU composites (**a**) Micrograph of the PUH sample (a*) Micrograph of the PUT sample (**b**) Micrograph of the PUH1 sample (b*) Micrograph of the PUT1 sample (**c**) Micrograph of the PUH5 sample (c*) Micrograph of the PUT sample.

The SEM micrographs of the PUT and PUH series are shown in Figure 7. The morphological behavior is also one of the key factors in elaborating the possible arrangement of the constituents. The SS of the PU composites was observed as a dense portion, while the bright portion appeared due to the HS of the samples. A very clear globular structure was observed for the PUH pristine sample (a), whereas slightly large globules of the same type were observed for PUT samples (a*). By inducing the 1% concentration of chitosan, the slightly larger globules appeared (b), whereas the fibrils were observed in the case of PUH1 (b*), and more condensed fibrils were observed in the case of c and c*.

### 2.7. Conclusions

The persistent intake of biocompatible material to develop biocompatible composites has helped maintain an array of eco-friendly composites. The chitosan-based polyurethane elastomers were prepared by the reaction of polyol and diisocyanate. Chitosan was reinforced with the matrix to develop the biocomposites of polyurethane. For this purpose, chitosan was dispersed in the polyurethane matrix, and different series of polyurethane elastomers were synthesized by varying the concentration of reinforcement. The controlled samples of polyurethane films of different thicknesses were prepared, and the concentration of reinforcement was optimized systematically. The samples were characterized by using analytical techniques, such as FTIR, SEM, TGA, and DMA, while the water absorption capability was observed by the swelling test. All the samples of chitosan-based PU elastomers were examined structurally, morphologically, thermally, and mechanically by these techniques. Using the FTIR, different functional groups, like the OH group, were identified in the range of 3470 cm^−1^ to 3300 cm^−1^. Also, the urethane groups in the samples were observed by FTIR. While increasing the weight percentage of chitosan in the films, the TGA showed thermal stability at different temperatures ranging from 100 to 600 °C. The samples with a greater concentration of chitosan showed greater thermal stability as compared to blank film. The DMA indicated better mechanical properties for aliphatic diisocyanate-based polyurethane as compared to aromatic diisocyanate-based polyurethane composites. The morphological characterization was carried out by using SEM analysis, which showed that samples with chitosan favor the synthesis of ordered structure. Meanwhile, the degree of absorption and swelling results indicated that chitosan-based polyurethane elastomers showed inferior swelling properties as compared to blank film of PU. This work is basically a step towards the generation of original biocompatible materials, preferably useful for biomedical applications.

## 3. Experimental

### 3.1. Chemical

The chitosan-reinforced PU composites were synthesized by using the following chemicals in pure form. Polycaprolactone (CAPA) Mn = 2000 g-mol^−1^ (Sigma Aldrich, St. Louis, MI, USA), toluene diisocyanate (TDI) (Sigma Aldrich, St. Louis, USA), hexamethylene diisocyanate (HMDI) (Sigma Aldrich, St. Louis, MI, USA), 1, 4 butane diol (BD) (Sigma Aldrich, St. Louis, MI, USA), chitosan with low molecular weight and degree of quaternization > 50% (Sigma Aldrich, St. Louis, MI, USA), and acetone (Sigma Aldrich, St. Louis, MI, USA). In order to avoid moisture and other impurities, the vacuum demoisturization of diols was performed at 70 °C for 24 h. No further treatment was performed for the analytical-grade chemicals and reagents.

### 3.2. Fabrication of Chitosan-Reinforced Polyurethane Composites

**Figure 8:**
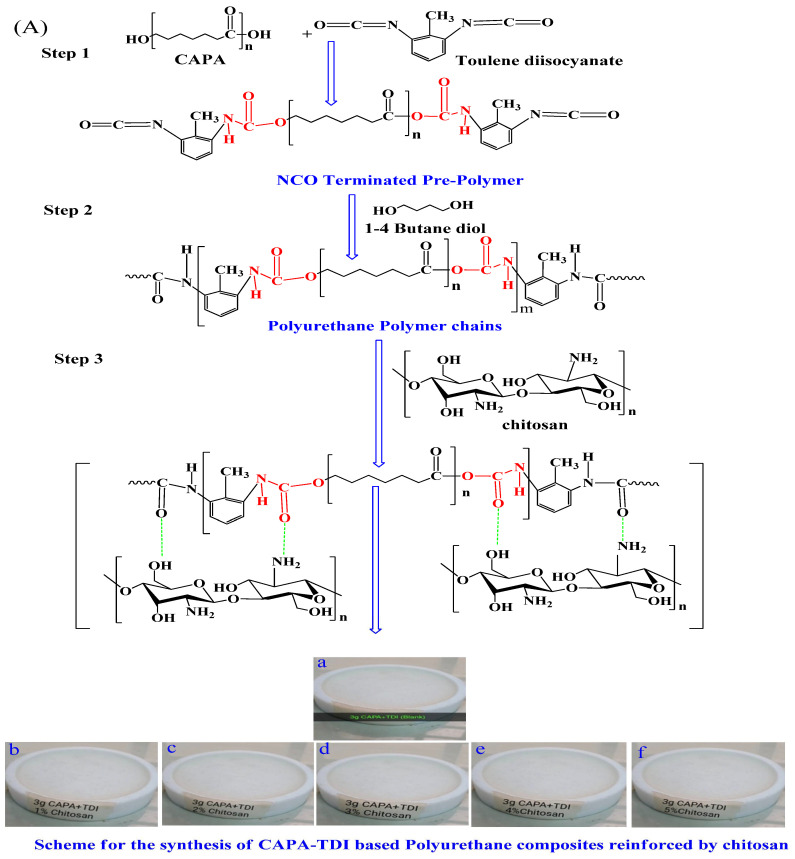

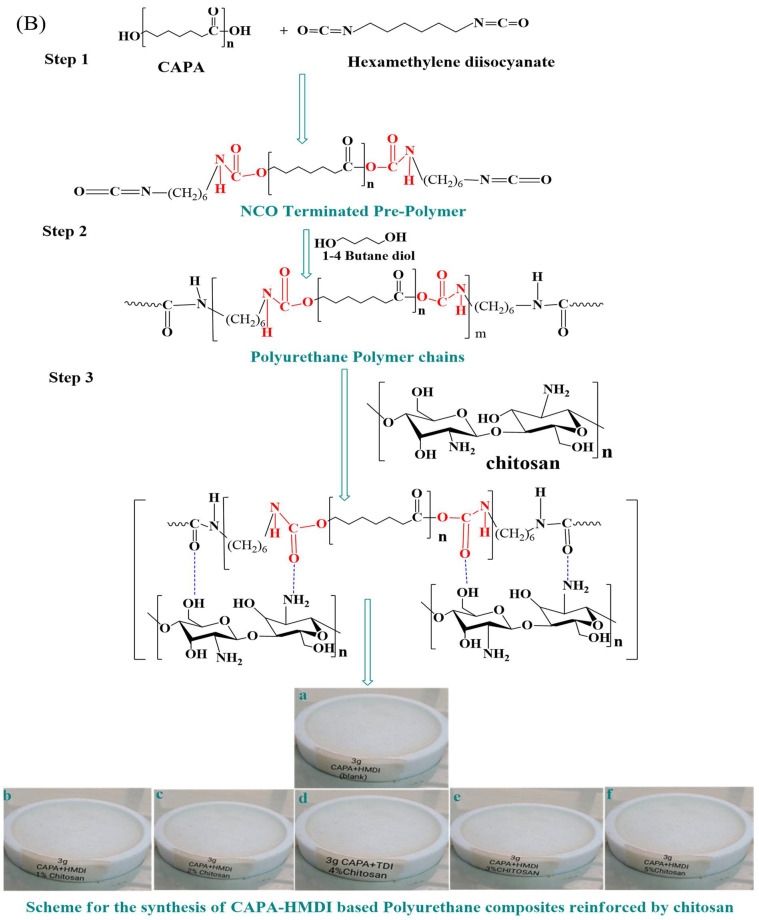
Synthetic route for the synthesis of PU composites (**A**) PU composites of the PUT series; step 1 is the representation of the prepolymer step; step 2 is the representation of PU chain formation; step 3 is the representation of the reinforcement of chitosan. Samples a–f show the synthesized samples of blank and 1–5% concentration of reinforcement. (**B**) PU composites of the PUH series; step 1 is the representation of the prepolymer step; step 2 is the representation of PU chain formation; step 3 is the representation of the reinforcement of chitosan. Samples a–f show the synthesized samples of blank and 1–5% concentration of reinforcement.

The fabrication of pristine PU samples and the chitosan-reinforced composites was carried out by using in situ polymerization. The details of the synthetic procedure are mentioned in our previous publications [4,20,41]. The experimental setup for the synthesis of PU is simple yet inevitable. The fabrication process itself consists of several vital steps and conditions. The initial step of PU fabrication was performed in a conventional round bottom flask amended with four openings/necks. These necks were used for the quick fitting of some essential apparatus, such as a mechanical stirrer for the stirring of monomers, a thermometer to maintain and record the temperature, a reflux condenser to maintain the temperature of the assembly, and N_2_ gas supply for the expulsion of oxygen. The pyrogallol solution was used to maintain the stream of nitrogen flow at the rate of 10–12 bubbles/min. The reaction temperature was maintained throughout the reaction with the aid of an oil bath. During the first step of the polymer’s fabrication, the stoichiometrically mandatory volume of diisocyanate was added to the reaction flask and the temperature was maintained up to 60 °C. The macrodiol CAPA was dispensed in the diisocyanate in the required quantity while the temperature was increased up to 100 °C. The unremitting stirring developed NCO-terminated PU prepolymer in approximately one hour. For the termination of NCO-ended chains, vigorous stirring was carried out using BD, resulting in the conversion of NCO-terminated prepolymer chains into the final product. In order to maintain the consistency of the fluid, the chain termination process proceeded for 15 min with the aid of acetone (20 wt %). The pristine PU sample was obtained by lowering the reaction mixture temperature. The fabrication of composites was carried out by the addition of chitosan in the stochiometric ratio. Chitosan was used in concentrations from 1–5 (wt %), whereas the hard segment contents were maintained up to 30 (wt %). The reinforcing agent chitosan was suspended in accordance with the optimization standards of protocols, including temperature, reaction stirring speed, and atmosphere of the fabrication. In order to avoid the interference of the atmospheric oxygen, the reaction was carried out by continuous purging of nitrogen gas. In order to attain the consistent mixing of chitosan, vigorous stirring was sustained at 1300 rpm with the help of a mechanical stirrer. Polytertaflouroethylene (PTFE) molds were used to cast the fabricated samples. The degassing at 50 °C was carried out under vacuum for 8 h until the evaporation of solvent took place smoothly. The ultimate sample drying proceeded gradually at different temperatures and different time intervals. In the first step of drying, the film was desiccated at 70 °C for 36 h. In the second step, drying was performed at 110 °C for 4 h. The film thickness of the dried composites was maintained at 2 ± 0.05 mm. All the fabricated composites were stored in a desiccator under ambient conditions for analysis. A similar procedure was followed to fabricate two series of composites. Each series contained six samples, including a pristine sample of PU. An inclusive detail of the fabricated samples in terms of stoichiometry is provided in Table 3. The schemes for the fabrication of two series of PU composites are illustrated in Figure 8A,B. All the fabricated samples were subjected to FTIR, TGA, DMA, and SEM for structural, thermal, mechanical, and morphological characterizations. The water retention tendency was observed using the swelling test. The biocompatibility of the fabricated samples was evaluated using hemolytic activity and the inhibition of biofilm formation against *E. coli* and *S. aureus*. The results of biofilm inhibition were also supported by phase contrast microscopy.

### 3.3. Characterization

#### 3.3.1. Fourier-Transform Infrared Spectroscopy (FTIR)

The FTIR analyses were performed on all the samples of PU composites by using a NICOLET 6700 spectrometer. The FTIR was attached with attenuated total reflectance (ATR), which was used as an accessory. The scans of the analyses were recorded in transmission mode (%) in the range of 4000–400 cm^−1^.

#### 3.3.2. Thermal Gravimetric Analysis (TGA)

The thermogravimetric curves of all PU composites were recorded using Perkin Elmer’s Thermogravimetric Analyzer (TGA) (Waltham, MA, USA). The ramp rate of 10 °C min^−1^ was used for the temperature range of 50–600 °C under N_2_ atmosphere with a flow rate of 50 mL/min [4].

#### 3.3.3. Dynamic Mechanical Analysis (DMA)

The DMA curves of PU composites were recorded on DMA (Q800; TA Instruments, New Castle, DE, USA). For the recording of data, the temperature sweep mode was used. The values of the storage modulus (E′), loss modulus (E″), and complex modulus (E*) were recorded. The DMA analysis was performed on the following dimensions: length; 50 mm, width; 2.5 mm, Thickness 1–2 ± 0.5 mm [20].

#### 3.3.4. Swelling Test

The polarity of the synthesized PU composites was evaluated by performing the solvent intake experiment. The PU composites were waterlogged in distilled H_2_O to evaluate the disparity in their weight change (%) until a constant weight was documented. The swelling was calculated using Equation (1) [47].

(1) $$\%water\;absorption=\frac{wet\;weight-Dry\;weight}{Total\;weight}\times 100$$

#### 3.3.5. Scanning Electron Microscopy (SEM)

A few selected samples of PU composites were subjected to scanning electron microscopic analysis. The surface morphology of the selected PU composites was performed with the help of JEOL JSM-7000F, with an accelerating voltage of 20 kV and a distance of 9.0 ± 0.5 mm.

#### 3.3.6. Biological Activity Tests

##### Hemolytic Activity

The hemocompatibility of the PU composites was executed [42,47,48,49,50] with the help of a polystyrene-based screw-cap tube. The tube was filled with freshly heparinized human blood (5 mL). Centrifugation was performed on this blood suspension for 5.0 min at 850 rpm. The supernatant was discarded. Phosphate buffer saline (PBS) (pH~7.4) was used to wash the sticky pellets. The washing was performed at least three times before leaving the pellets in PBS (20 mL). In order to perform the experimental protocol, a small portion of PU composite (20 μL) was added into the microfuge tube (2.0 mL). The homogenous mixing of cell suspensions (180 μL) was performed. For the analysis, PBS was used as a negative control, whereas the triton X-100 (0.1%) was used as a positive control. The Eppendorf tubes were centrifuged for a short time (10 min). The supernatant (100 μL) was placed in a sterilized microfuge tube and diluted with PBS (900 μL). The suspension was divided into three replicates (200 μL). The supernatant was placed into a sterile 96-well microplate comprising one positive and one negative control. Absorbance was registered at 540 nm on a microplate reader (BioTek, Winooski, VT, USA)). Equation (2) was used to calculate the hemolytic value (%) of each PU composite sample [8].

(2) $$Hemolysis(\%)=\frac{Sample\;absobance-negative\;control}{Positive\;control}\times 100$$

##### Biofilm Inhibition Assay

The biofilm inhibition activity was evaluated against *Staphylococcus aureus (S. aureus)* and *Escherichia coli (E. coli)*, as reported earlier [52,53]. A sterile plastic tissue culture 96-well plate was filled with nutrient broth (100 μL) (Oxoid, Basingstoke, UK) and each PU composite suspension (100 μL) was inoculated with bacterial suspension (20 μL) into the wells separately. Bacteria and its nutrient broth in wells were used as a control for both strains (10 μg/20 μL) without PU composite suspensions. Under aerobic conditions, the 96-well plate was covered and incubated at 37 °C (24 h). The positive standard (ciprofloxacin) was used to compare the results of PU composites. Incubation was followed by washing in triplicate with sterile phosphate buffer (220 μL to each well). The removal of non-adherent bacteria from wells was carried out by shaking the plate. After washing, in order to fix the attached bacteria, 99% methanol (220 μL per well) was added and stored (15 min), whereas methanol was later evaporated. For each well, staining was performed using 220 μL of 2% crystal violet (5 min). The excess stain was removed by washing the plate with water dried at room temperature. The re-solubilization of dye took place using 220 µL of 33% (*v*/*v*) glacial acetic acid in each well. The microplate reader (BioTek, Winooski, VT, USA) was used to measure the optical density for each well at 630 nm. Equation (3) was used to record the bacterial growth inhibition (INH%) [8].

(3) $$1NH(\%)=1-\frac{OD630\left(sample\right)}{OD630\left(control\right)}\times 100\%$$

##### Biofilm Inhibition through Phase Contrast Microscopy

Phase contrast microscopy was used to observe the biofilm inhibition aptitude of PU composites [43,54]. The experiment was performed using a few drops of *S. aureus* and *E. coli*, which were cultured on glass slides. The incubation was carried out at 37 °C for 14 h. The PBS solution was used for the washing of PU composite slides. After rinsing and staining the slides, the biofilms were dissolved using 30% glacial acetic acid. The controlled slides of samples, negative PBS control, and positive control with ciprofloxacin were developed without PU composite suspensions. The slides of all the suspensions were inspected microscopically.

## Figures and Tables

**Table 1:** A comprehensive profile of PU composites.

Sample Code	HS Contents ^a^ (wt %)	Optimum Swelling (%)	T @10% wt Loss (°C)	T @90% wt Loss (°C)	Complex Modulus′@25 °C (MPa)	Sample Code	HS Contents ^a^ * (wt %)	Optimum Swelling (%)	T @10% wt Loss (°C)	T @90% wt Loss (°C)	Complex Modulus′@25 °C (MPa)
PUT	5.0	10.0 ± 0.9	351	494	83	PUH	5.0	8.5 ± 0.5	380	506	236
PUT1	10.0	11.1 ± 1.0	353	497	46	PUH1	10.0	12.5 ± 0.5	246	415	3.36
PUT2	15.0	11.0 ± 1.0	384	503	135	PUH2	15.0	16.6 ± 0.4	311	374	2.73
PUT3	20.0	25.0 ± 0.8	388	518	221	PUH3	20.0	25 ± 0.3	362	488	57.5
PUT4	25.0	28.5 ± 1.0	386	517	322	PUH4	25.0	28.5 ± 0.2	317	504	64.9
PUT5	30.0	33.3 ± 1.0	403	510	64	PUH5	30.0	33.3 ± 1.0	286	454	50.9
**Significant FTIR Peaks**											
N-H (str) 3400–3325	CH_2_ (sym) 2882–2867	CH_2_ (Asym) 2954–2882	C=O (str) 1740–1709	N-H (bend) 1631–1549	C-H (wag) 960–940	C-O 1124–1096	W C–H (aromatic) 814–675	υC–O–C 1060–1049	υC–N 1231–1220	H–C=O 1716–1700	δC–H (aromatic) 1224–963

a = Hard Segment Content [21,22] = [(W_TDI_ + W_BD_ + W_chitosan_)/W_Total_] × 100. a* Hard Segment Content [21,22] = [(W_HMDI_ + W_BD_ + W_chitosan_)/W_Total_] × 100.

**Table 2:** Data of biocompatibility of polyurethane composites as hemolytic activity and inhibition of biofilm formation.

Sample Code	Hemolytic Activity (%)	Inhibition of Biofilm Formation against *E. coli* (%)	Inhibition of Biofilm Formation against *S. aureus* (%)	Sample Code	Hemolytic Activity (%)	Inhibition of Biofilm Formation against *E. coli* (%)	Inhibition of Biofilm Formation against *S. aureus* (%)
PUT	1.0 ± 0.5	1.4 ± 0.4	3.3 ± 0.8	PUH	1.7 ± 1.0	2.4 ± 0.5	3.3 ± 0.7
PUT1	8.2 ± 1.0	19.3 ± 1.0	20.2 ± 1.3	PUH1	5.2 ± 1.0	10.0 ± 1.0	13.5 ± 1.0
PUT2	10.0 ± 1.0	24.6 ± 1.2	24.6 ± 1.4	PUH2	8.2 ± 0.8	14.4 ± 1.0	19.3 ± 1.3
PUT3	10.5 ± 0.9	25.6 ± 1.5	36.2 ± 1.8	PUH3	9.4 ± 0.9	40.5 ± 1.4	27.0 ± 1.5
PUT4	11.1 ± 1.0	35.2 ± 1.5	37.6 ± 1.9	PUH4	14.1 ± 1.2	41.5 ± 1.5	29.9 ± 1.7
PUT5	12.3 ± 1.5	45.8 ± 1.8	42.0 ± 1.9	PUH5	15.8 ± 1.5	46.3 ± 1.8	46.3 ± 1.8
Triton X-100(Positive Control)	94.1 ± 0.28	-	-	Triton X-100(Positive Control)	94.1 ± 0.28	-	-
PBS(Negative control)	0.41 ± 0.10	-	-	PBS(Negative control)	0.41 ± 0.10	-	-
Ciprofloxacin (positive control)	-	82.65 ± 2.74	77.57 ± 2.37			82.65 ± 2.74	77.57 ± 2.37

**Table 3:** A comprehensive description of sample codes and stoichiometry of polyurethane composites containing aromatic/aliphatic diisocyanate.

Series-I					Series-II			
Sample Code	Composition CAPA/TDI/BD 1:2:1 NCO/OH	Chitosan (%)	HS Contents ^a^ (wt %)	SS Contents ^b^ (wt %)	Sample Code	Composition CAPA/HMDI/BD 1:2:1 NCO/OH	Chitosan (%)	HS Contents ^a^* (wt %)
PUT	1/1	0.0	5.0	95	PUH	1/1	0.0	5.0
PUT1	1/1	1.0	10.0	90	PUH1	1/1	1.0	10.0
PUT2	1/1	2.0	15.0	85	PUH2	1/1	2.0	15.0
PUT3	1/1	3.0	20.0	80	PUH3	1/1	3.0	20.0
PUT4	1/1	4.0	25.0	75	PUH4	1/1	4.0	25.0
PUT5	1/1	5.0	30.0	70	PUH5	1/1	5.0	30.0

a = Hard Segment Content [21,22] = [(W_TDI_ + W_BD_ + W_chitosan_)/W_Total_] × 100. b = Soft Segment Content = [100 − Hard Segment Content (%)]. a* Hard Segment Content [21,22] = [(W_HMDI_ + W_BD_ + W_chitosan_)/W_Total_] × 100.

## Data Availability

The data presented in this study are available on request from the corresponding author.

## Sample Availability

Not available.
